# No Evidence for the Presence of SARS-CoV-2 in Bank Voles and Other Rodents in Germany, 2020–2022

**DOI:** 10.3390/pathogens11101112

**Published:** 2022-09-28

**Authors:** Kerstin Wernike, Stephan Drewes, Calvin Mehl, Christin Hesse, Christian Imholt, Jens Jacob, Rainer G. Ulrich, Martin Beer

**Affiliations:** 1 Institute of Diagnostic Virology, Friedrich-Loeffler-Institut, 17493 Greifswald-Insel Riems, Germany; 2 Institute of Novel and Emerging Infectious Diseases, Friedrich-Loeffler-Institut, 17493 Greifswald-Insel Riems, Germany; 3 German Centre for Infection Research (DZIF), Site Hamburg-Lübeck-Borstel-Riems, 17493 Greifswald-Insel Riems, Germany; 4 Rodent Research, Institute for Epidemiology and Pathogen Diagnostics, Julius Kühn-Institute (JKI), Federal Research Centre for Cultivated Plants, 48161 Münster, Germany

**Keywords:** coronavirus, COVID-19, reservoir, intermediate host, Cricetidae, Muridae

## Abstract

Rodentia is the most speciose mammalian order, found across the globe, with some species occurring in close proximity to humans. Furthermore, rodents are known hosts for a variety of zoonotic pathogens. Among other animal species, rodents came into focus when the severe acute respiratory syndrome coronavirus type 2 (SARS-CoV-2) spread through human populations across the globe, initially as laboratory animals to study the viral pathogenesis and to test countermeasures. Under experimental conditions, some rodent species including several cricetid species are susceptible to SARS-CoV-2 infection and a few of them can transmit the virus to conspecifics. To investigate whether SARS-CoV-2 is also spreading in wild rodent populations in Germany, we serologically tested samples of free-ranging bank voles (*Myodes glareolus*, n = 694), common voles (*Microtus arvalis*, n = 2), house mice (*Mus musculus*, n = 27), brown or Norway rats (*Rattus norvegicus*, n = 97) and *Apodemus* species (n = 8) for antibodies against the virus. The samples were collected from 2020 to 2022 in seven German federal states. All but one sample tested negative by a multispecies ELISA based on the receptor-binding domain (RBD) of SARS-CoV-2. The remaining sample, from a common vole collected in 2021, was within the inconclusive range of the RBD-ELISA, but this result could not be confirmed by a surrogate virus neutralization test as the sample gave a negative result in this test. These results indicate that SARS-CoV-2 has not become highly prevalent in wild rodent populations in Germany.

## 1. Introduction

The order Rodentia is a highly diverse group, encompassing more than 2000 species worldwide. Rodents, and representatives of the families Muridae and Cricetidae in particular, are well-known hosts for a variety of zoonotic pathogens [1]. Because of their global distribution and high abundance of some species in close proximity to humans in urban and suburban settings, rodents pose a considerable risk for the back-transmissions of human pathogens once established in these rodent populations. Given that two (OC43 and HKU1) of the seven currently known human coronaviruses have related ancestors in rodent species [2] and that additional rodent-specific coronaviruses have been described [3], it stands to reason that rodents also come into focus when investigating potential reservoir hosts for the severe acute respiratory syndrome coronavirus type 2 (SARS-CoV-2). SARS-CoV-2 was initially detected in late 2019 in Wuhan, China, where it caused an outbreak of an acute respiratory disease in humans [4]. The outbreak of the novel disease, now named COVID-19 (coronavirus disease 2019), very rapidly evolved into a global pandemic [5], resulting in millions of cases worldwide [6]. At the beginning of the pandemic, when vaccines and treatment options were not yet available, suitable animal models that reflect different aspects of the SARS-CoV-2 pathogenesis were urgently needed to assist in the development of countermeasures. Some rodent species, such as Norway or brown rats (*Rattus norvegicus*), house mice (*Mus musculus*) and golden hamsters (*Mesocricetus auratus*), are the most widely used laboratory animals in biomedical research and were investigated regarding their susceptibility to SARS-CoV-2 early into the pandemic (reviewed in [7]). Although house mice could be excluded, through experimental infection studies, as amplifying hosts for the wild-type virus [8], they are susceptible to some of the recently emerging variants of concern (VOCs) of SARS-CoV-2, in particular the beta (B.1.351) and gamma (P1) VOCs [9,10]. Furthermore, SARS-CoV-2 replication is supported in some Cricetidae species. Though the course of disease and the outcome of infection vary between the different hamster species investigated so far, all species tested are susceptible [11,12,13,14,15,16]. Alarmingly, recent reports show the first natural zooanthroponotic infections of humans by pet hamsters [17,18]. In addition to hamster, other Cricetidae species such as the bushy tailed woodrats (*Neotoma cinerea*), North American deer mice (*Peromyscus maniculatus*), white-footed mice (*P. leucopus*) and the Eurasian bank vole (*Myodes glareolus*) can be experimentally infected with SARS-CoV-2 [8,19,20,21]. Again, the exact course of infection may differ between these species, potentially dependent on the virus variant, but in general, the infectious virus is shed for several days, in some cases leading to infection of naïve animals through direct contact [8,20]. Hence, there is a potential for SARS-CoV-2 transmission to humans from infected rodents when the virus occurs at high prevalences in wild rodent populations. To characterize the risk for SARS-CoV-2 presence in free-ranging rodents in Germany, we serologically investigated samples collected in 2020, 2021 and 2022, with a special focus on the cricetid species bank vole. In total, 694 samples taken from bank voles in four German federal states, two samples from common voles (*Microtus arvalis*), 27 samples from house mice, 97 Norway rats and eight individuals of *Apodemus* species were tested.

## 2. Results

**Figure 1:**
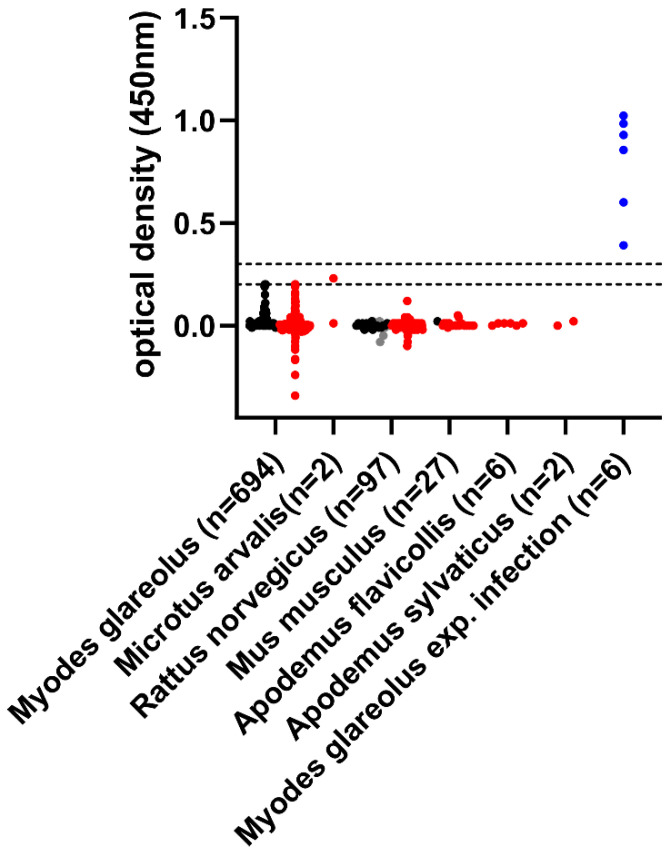
Results of the RBD-based SARS-CoV-2 ELISA. The cut-offs for negativity (≤0.2) and positivity (≥0.3) are indicated by horizontal dashed lines. The results of the samples collected until February 2021 are shown in black, while the results of all samples collected from March 2021 onwards, when different VOCs were circulating in the human population in Germany, are shown in red. For a few rat samples, the exact trapping date could not be identified; the results of these samples are depicted in grey. For comparison, the values obtained from blood samples collected on day 8 (two animals), 12 (one animal) or 21 (three animals) after experimental SARS-CoV-2 infection of bank voles [19] are included (shown in blue).

All but one of the 828 samples scored negative in a multispecies ELISA based on the receptor binding domain (RBD) of SARS-CoV-2, regardless of whether the wild-type virus or VOCs have been circulating in the human population during the sampling period (Figure 1). The remaining sample (lavage of a common vole collected in 2021 in Baden-Wuerttemberg) resulted in an inconclusive value within the measuring range of the RBD-ELISA; however, the result could not be confirmed since the sample tested clearly negative by a SARS-CoV-2 surrogate virus neutralization test (5.6% inhibition, cut-off for positivity at ≥30% inhibition).

## 3. Discussion

The human SARS-CoV-2 pandemic, which has now been going on for more than 2.5 years and incurred hundreds of millions of infected individuals worldwide during that time [6], is presently driven by direct human-to-human virus transmission via aerosolized particles. However, ever since the beginning of the pandemic the role of animals as potential intermediate or reservoir hosts has been discussed. When the virus was introduced into mink (*Neovison vison*) farms and caused local epidemics in these highly susceptible animal species [22,23,24,25], evidence for mink-to-human spillback infections was reported [26], resulting in concerns about viral maintenance and evolution/adaptation in animals. Aside from these mink-to-human and the likely initial animal-to-human transmissions, suspicions of zooanthroponotic SARS-CoV-2 transmissions were reported from a cat (*Felis catus*) and pet hamsters [17,18,27], expanding the species potentially representing a risk for infections of humans to companion animals and rodents in particular.

Here, we focused mainly on bank voles as they are widely distributed and can occur in high densities in nature, are well-known reservoir hosts for zoonotic agents and belong, like hamster, to the Cricetidae family within the order Rodentia. Under experimental conditions, intranasal inoculation of bank voles with wild-type SARS-CoV-2 led to viral replication in the respiratory tract, without inducing any obvious clinical signs, followed by seroconversion [19]. In addition, low amounts of viral RNA could be detected in the central nervous and lymphatic systems [19]. Intra-species transmission to direct contact animals, which would be needed to establish effective transmission cycles in nature, was not observed [19], but information about the effect of SARS-CoV-2 VOCs on bank voles and any potential alteration of the infection characteristics are currently missing. For house mice, precisely those alterations could be seen as wild-type house mice are not susceptible to the wild-type virus, but can be infected with some of the recently emerging VOCs of SARS-CoV-2 [9,10]. However, the VOCs for which a higher susceptibility of rodents was seen, i.e., the beta and gamma variants, never exceeded a proportion of 4% (beta and gamma combined) in the human population in Germany [28]. In humans, the wild-type virus was dominant in Germany until the end of February 2021, when a shift to alpha (B.1.1.7) as the dominant variant occurred, followed by delta (B.1.617.2) that took over the dominance in calendar weeks 24/25 of 2021. Since the turn of the year, from 2021 to 2022, omicron (B.1.1.529) and its diverse sub-variants was the predominant variant detected in human patients [28]. Hence, in the sampling period of our study, mainly virus variants for which rodents show a lower susceptibility were present in the human population, which might have influenced the transmission potential to wild rodents and, as a consequence, the lack of SARS-CoV-2 in the surveilled populations. Nevertheless, despite the consistently negative serological results in our study, it is recommended to include rodents also in further monitoring efforts.

Other rodent species that live in high-density settings in close proximity to humans and that are known hosts of other (non-SARS-CoV-2) coronaviruses are Norway rats and house mice [29,30,31]. Both species can be efficiently infected by, e.g., the beta variant of SARS-CoV-2 [32]. In addition, the virus can be transmitted from infected Norway rats, with moderate efficiency, to conspecifics through direct contact [32], making the establishment of effective infection chains in nature likely. During a recent study, Norway rats inhabiting the sewer system of Antwerp, Belgium, were investigated for SARS-CoV-2 RNA or antibodies against the virus and all tested negative [33]. That sampling location was selected because SARS-CoV-2 was repeatedly detected in wastewater [34], thereby representing a possible route of exposure for rats. Though only a limited number of rats (<40) were tested by serology or PCR methods [33], the consistently negative results suggest a low probability of virus introduction into the surveilled urban rat population. In our study, 97 rat samples, collected in a different country in the context of pest control, were tested and all of them were likewise seronegative, confirming the low probability of virus spread in free-ranging Norway rats, at least for the SARS-CoV-2 variants circulating in humans during the study period.

In conclusion, we found no evidence for the presence of SARS-CoV-2 in the surveilled wild rodent populations using serological test systems that have been demonstrated to detect antibodies against diverse VOCs of SARS-CoV-2 [35,36]. Nevertheless, rodents should be included in future monitoring studies, especially in regions with high human population densities and where the beta and gamma variants are more prevalent, and when new virus variants emerge during the continued evolution of SARS-CoV-2 and when the potential of the variants to break cross-species barriers and the capability to expand species tropism to animals is unknown.

## 4. Materials and Methods

A total of 694 samples collected from bank voles in 2020 and 2021 in four German federal states (Baden-Wuerttemberg, Brandenburg, Lower Saxony, North Rhine-Westphalia) were tested (Table 1). The human population density in these federal states ranges from 86 (Brandenburg) to 525 (North Rhine-Westphalia) inhabitants per square kilometre (https://de.statista.com/statistik/daten/studie/1242/umfrage/bevoelkerungsdichte-in-deutschland-nach-bundeslaendern/, accessed on 19 September 2022). The samples that were collected in 2020 for a bank vole monitoring study are described in [37]. The sample matrices comprised blood (n = 614) collected during live-trapping and chest cavity lavage fluid from animal carcasses from snap trapping (n = 80). The latter was collected by rinsing the chest cavity with 1 mL phosphate-buffered saline (PBS) during necropsy. Additional rodents were collected during pest control in different zoological gardens and an agricultural company. Lavages of 27 house mice, 97 Norway rats, two common voles and eight individuals of the genus *Apodemus* were included, the origin of the samples is given in Table 1.

All samples were tested by a SARS-CoV-2 RBD-based multispecies ELISA carried out as described previously [38]. Blood samples were pre-diluted 1/100 as described and lavage samples were tested in a 1/10 dilution. During the initial test validation, a cut-off of ≤0.2 was set for negativity and ≥0.3 for positivity, with the intermediate zone between 0.2 and 0.3 being inconclusive [38]. The suitability of the indirect ELISA for the investigation of rodent sera was validated by testing samples of experimentally SARS-CoV-2 infected animals (bank voles and house mice) [19,39] or of vaccinated animals (Norway rats, unpublished). As positive controls, blood samples collected on day 8 (two animals), 12 (one animal) or 21 (three animals) after experimental SARS-CoV-2 inoculation of bank voles [19] have been tested, the results are shown in Figure 1. The suitability of the ELISA to detect antibodies directed against diverse VOCs has been shown during experimental infection studies [35,36].

Samples that tested positive or inconclusive in the RBD-ELISA were subsequently tested by a surrogate virus neutralization test (cPass SARS-CoV-2 Surrogate Virus Neutralization Test (sVNT) Kit, GenScript, Rijswijk, the Netherlands) performed as prescribed by the manufacturer (cut-off ≥ 30% positive and <30% negative). The surrogate virus neutralization test in its original composition detects antibodies against the wild-type virus and all VOCs except omicron. For omicron (and its diverse sub-variants), a specific horseradish peroxidase (HRP)-conjugated RBD is provided by the test manufacturer. As the sample that resulted in an inconclusive value within the measuring range of the in-house RBD-ELISA was collected in 2021, i.e., prior to the emergence of omicron in the study area, we applied the original composition of the surrogate virus neutralisation test.

## Figures and Tables

**Table 1:** Rodent samples that were tested for the presence of antibodies against SARS-CoV-2.

Rodent Species	Sample Material	Federal State	Year	Number of Samples
*Myodes glareolus*	blood	NW	2021	614
*Myodes glareolus*	chest cavity lavage	BB, BW, NI	2020/21	80
*Microtus arvalis*	chest cavity lavage	BW	2021	2
*Mus musculus*	chest cavity lavage	HE, SH	2021/22	27
*Rattus norvegicus*	chest cavity lavage	BB, BW, MV, SH	2020/21/22	97
*Apodemus flavicollis*	chest cavity lavage	BW, SH	2021/22	6
*Apodemus sylvaticus*	chest cavity lavage	BW, HE	2021	2

NW—North Rhine-Westphalia, BB—Brandenburg, BW—Baden-Wuerttemberg, NI—Lower Saxony, HE—Hesse, SH—Schleswig-Holstein, MV—Mecklenburg-Western Pomerania.

## Data Availability

The data presented in this study are available in the article.
